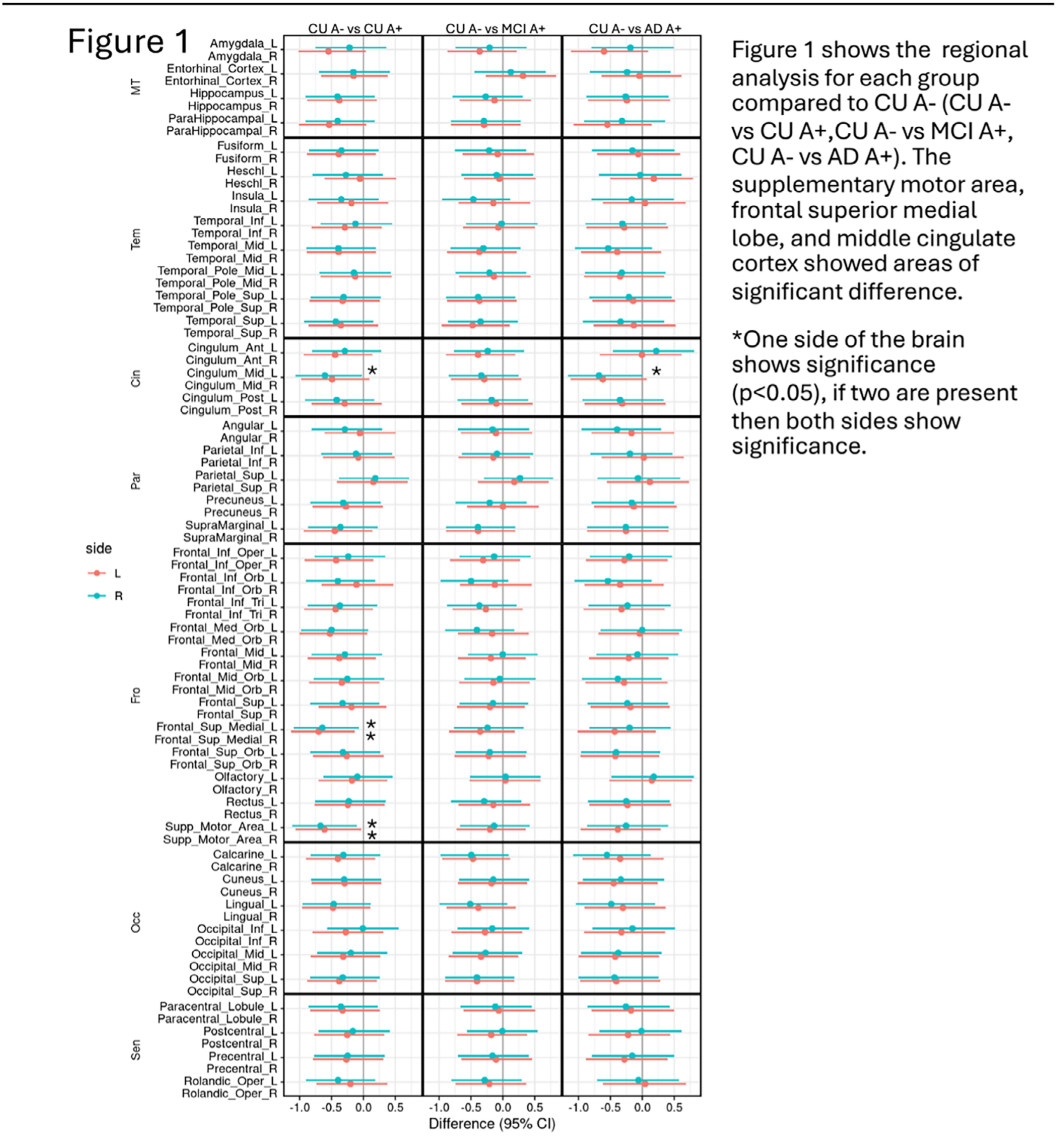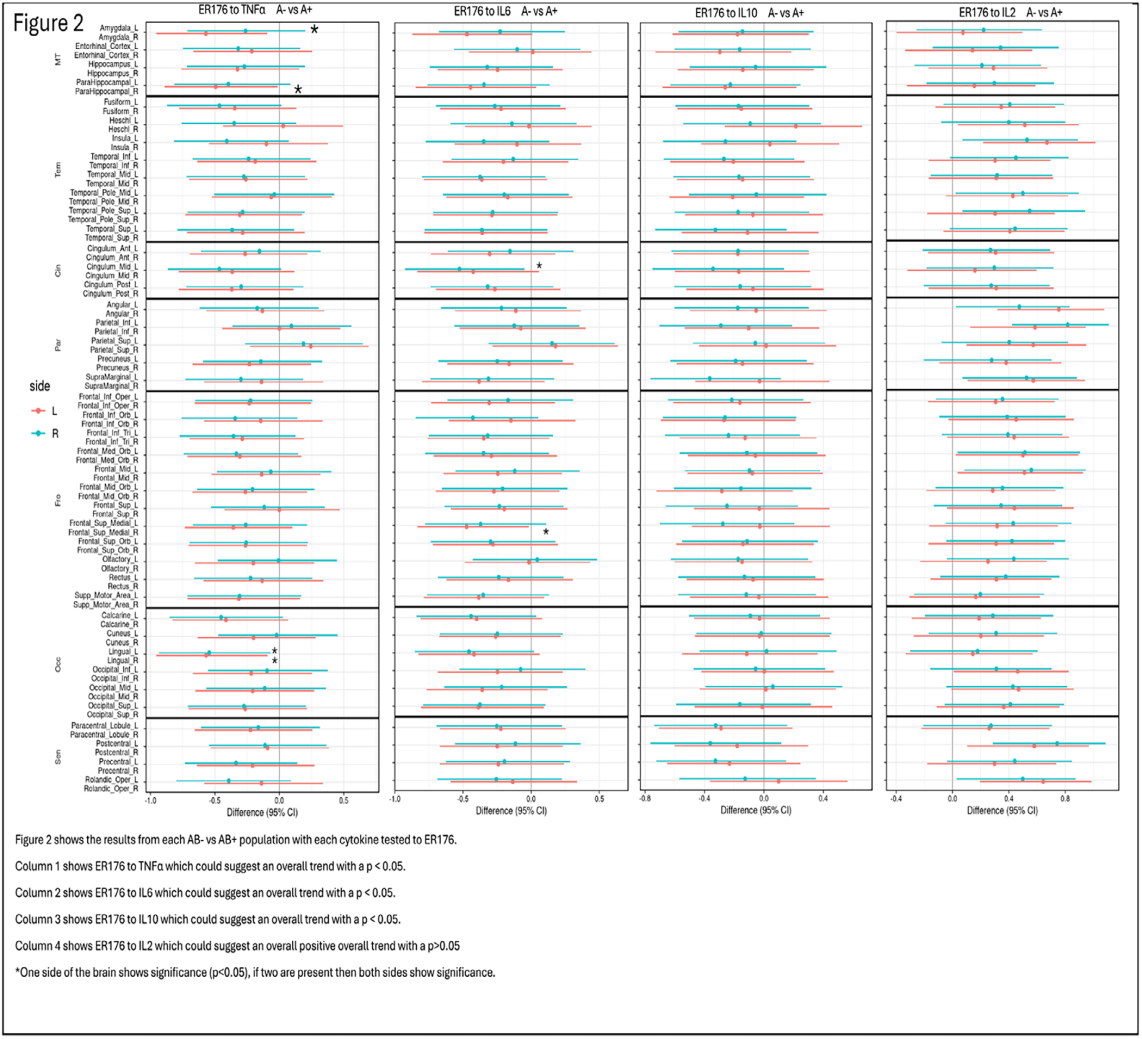# Assessing the relationship of systemic proinflammatory biomarkers in the Alzheimer's disease cognitive spectrum using ER176 neuroinflammation PET

**DOI:** 10.1002/alz70855_106903

**Published:** 2025-12-24

**Authors:** David N Jacobson, Mahathi Kandimalla, Seokbeen Lim, Emily S. Lundt, Jeyeon Lee, Paul H Min, Hugo Botha, Jonathan Graff‐Radford, David T. Jones, Prashanthi Vemuri, Kejal Kantarci, David S. Knopman, Clifford R. Jack, Ronald Petersen, Val J Lowe

**Affiliations:** ^1^ Mayo Clinic, Rochester, MN, USA; ^2^ Hanyang University, Seoul, Korea, Republic of (South); ^3^ Department of Neurology, Mayo Clinic, Rochester, MN, USA; ^4^ Department of Radiology, Mayo Clinic, Rochester, MN, USA

## Abstract

**Background:**

Some data suggest that neuroinflammation may be associated with pro‐inflammatory states in the body. Systemic, proinflammatory cytokines (TNFα, IL‐1, IL‐6, etc.) are produced by a variety of comorbidities. Neuroinflammation has been shown to be associated with the accumulation of amyloid in the brain (Becher, Heneka). We hypothesized that systemic cytokine production could be associated with neuroinflammation and thereby be related to brain amyloid accumulation. This study aims to evaluate the relationship between neuroinflammation, amyloid accumulation and systemic cytokines.

**Method:**

90 participants from the Mayo Clinic Study of Aging (MCSA) received a fasting cytokine panel over a 5‐year span where IL‐1, IL‐6, IL‐10, TNFa levels were measured. Participants were divided into four groups: cognitively unimpaired with normal levels of amyloid (CU A‐, n = 25), cognitively unimpaired with elevated amyloid (CU A+, n = 25), and mild cognitively impaired (MCI) with amyloid positive (MCI A+, n = 25), and Alzheimer's disease (AD) with elevated amyloid (AD A+, n = 15). To compare regional standardized uptake value ratio (SUVr) from neuroinflammation PET based on amyloid status (A+ vs A‐) and the four cytokines, Spearman's rank correlation and Fishers r‐to‐z transformations were used to assess pairwise group differences.

**Result:**

Using Spearman's rank correlation and Fishers r‐to‐z transformations, no strong relationship was found between systemic inflammation and neuroinflammation. While comparing CUA‐ vs CUA+ in Figure 1, three regions (supplementary motor area, frontal superior medial lobe, and middle cingulate cortex) showed a significant difference (*p* < 0.05). However, Figure 2 showed an overarching trend bias towards significance across most regions in IL10, IL6, and TNFa.

**Conclusion:**

Although significant relationships between systemic inflammation, brain amyloid and neuroinflammation were limited, the data show an overarching trend bias in broad brain regions, suggesting that further analyses are needed, possibly with larger sample sizes, to assess the relationship of regional neuroinflammation and amyloid to systemic inflammation. Future analyses could also regress out factors like sex, age, and genotype to further proinflammatory cytokine research.